# The hybrid non-ethylene and ethylene ripening response in kiwifruit (*Actinidia chinensis*) is associated with differential regulation of *MADS*-box transcription factors

**DOI:** 10.1186/s12870-015-0697-9

**Published:** 2015-12-29

**Authors:** Peter A. McAtee, Annette C. Richardson, Niels J. Nieuwenhuizen, Kularajathevan Gunaseelan, Ling Hoong, Xiuyin Chen, Ross G. Atkinson, Jeremy N. Burdon, Karine M. David, Robert J. Schaffer

**Affiliations:** The New Zealand Institute for Plant & Food Research Limited (PFR), Mt Albert Research Centre, Auckland, New Zealand; School of Biological Sciences, University of Auckland, Auckland, New Zealand; PFR, Kerikeri Research Station, Kerikeri, New Zealand

**Keywords:** *Actinidia*, Fruit Ripening, Ethylene, Ripening Inhibitor

## Abstract

**Background:**

Ripening in tomato is predominantly controlled by ethylene, whilst in fruit such as grape, it is predominantly controlled by other hormones. The ripening response of many kiwifruit (*Actinidia*) species is atypical. The majority of ripening-associated fruit starch hydrolysis, colour change and softening occurs in the apparent absence of ethylene production (Phase 1 ripening) whilst Phase 2 ripening requires autocatalytic ethylene production and is associated with further softening and an increase in aroma volatiles.

**Results:**

To dissect the ripening response in the yellow-fleshed kiwifruit *A. chinensis* (‘Hort16A’), a two dimensional developmental stage X ethylene response time study was undertaken. As fruit progressed through maturation and Phase 1 ripening, fruit were treated with different concentrations of propylene and ethylene. At the start of Phase 1 ripening, treated fruit responded to ethylene, and were capable of producing endogenous ethylene. As the fruit progressed through Phase 1 ripening, the fruit became less responsive to ethylene and endogeneous ethylene production was partially repressed. Towards the end of Phase 1 ripening the fruit were again able to produce high levels of ethylene. Progression through Phase 1 ripening coincided with a developmental increase in the expression of the ethylene-unresponsive MADS-box *FRUITFUL-*like gene (*FUL1*). The ability to respond to ethylene however coincided with a change in expression of another MADS-box gene *SEPALLATA4*/*RIPENING INHIBITOR*-like (*SEP4/RIN*). The promoter of *SEP4/RIN* was shown to be transactivated by EIN3-like transcription factors, but unlike tomato, not by SEP4/RIN itself. Transient over-expression of *SEP4/RIN* in kiwifruit caused an increase in ethylene production.

**Conclusions:**

These results suggest that the non-ethylene/ethylene ripening response observed in kiwifruit is a hybrid of both the tomato and grape ripening progression, with Phase 1 being akin to the *RIN*/ethylene inhibitory response observed in grape and Phase 2 akin to the *RIN*-associated autocatalytic ethylene response observed in tomato.

**Electronic supplementary material:**

The online version of this article (doi:10.1186/s12870-015-0697-9) contains supplementary material, which is available to authorized users.

## Background

In all fleshy fruits, fruit maturation and ripening is achieved through complex metabolic processes that are regulated by both developmental and hormonal factors. While hormones such as auxin, abscisic acid and cytokinins have all been linked to the ripening process [1], the best characterised hormone is ethylene, due to its extreme ripening effect in many fruit. Ethylene is synthesised in a simple three-step pathway from methionine, through *S-ADENOSYL METHIONINE SYNTHETASE* (*SAM*), *1-AMINO CYCLOPROPANE-1-CARBOXYLATE SYNTHASE* (*ACS*) and *ACC OXIDASE* (*ACO*) [2]. All these genes are associated with multi-gene families, and in many plants *ACS* has been shown to be a rate-limiting step for ethylene biosynthesis [2]. The *ACS* gene family consist of three classes of genes, depending on the presence of destabilisation elements in the carboxy (C) termini [3] that are regulated by the F-box genes *ETHYLENE OVER PRODUCER* (*ETO*) [4]. In fruit with autocatalytic ethylene-associated ripening, specific members of each of these biosynthetic gene-families are associated with ripening, which, when suppressed result in a loss or reduction in ripening is observed [5–8].

Fruit perceive ethylene through a multi-step signalling pathway that begins with the binding of ethylene to a two-component transmembrane receptor complex found at the endoplasmic reticulum [9–12]. Multiple ethylene receptor and ethylene sensor complexes have been identified in tomato and *Arabidopsis* that have also been shown to bind ethylene. The binding of ethylene to these receptors suppresses a mostly linear pathway that ultimately leads to the stabilisation of the EIN3 family of transcription factors which regulate ethylene-responsive genes, reviewed [13]. Transcription factors such as the *SQUAMOSA BINDING PROTEIN* (*SQBP*), *COLOURLESS NON RIPENING* (*CNR*) [14], *ETHYLENE RESPONSE FACTORS* (*ERF*), as well as various MADS-box genes regulate downstream ripening genes [15, 16]. In tomato, strawberry, apple, banana and grape, maturation and pre-ethylene ripening events have also been associated with the MADS-box transcription factor class of genes linked to floral organ identity. These include the *SEPALLATA* (*SEP*)-like *RIPENING INHIBITOR* (*RIN*) [17–20], *FRUITFUL* (*FUL*)-like *TDR4* [21] and the *AGAMOUS* (*AG*)-like *TAGL1* [22–24], which interact with one another to switch on ripening-associated genes such as those involved in ethylene biosynthesis [25–29].

Ripening of yellow-fleshed *Actinidia chinensis* ‘Hort16A’ fruit is a complex and a highly co-ordinated process that involves changes in flesh texture [30] and colour [31], conversion of starch to soluble carbohydrates [32, 33] and the development of taste and aroma compounds [34, 35]. A major ripening change in *Actinidia* spp. is textural, from a firm texture (>50 Newton (N) firmness) to a soft melting texture (<10 N firmness). The softening of kiwifruit to eating ripeness (6-8 N firmness) occurs largely in the absence of any detectable ethylene [33, 36, 37], although firm fruit may be extremely responsive to exogenously applied ethylene [32]. This capacity to respond to ethylene develops progressively in the fruit whilst on the vine [32, 38]. When mapped to the phenological Biologische Bundesanstalt, Bundessortenamt und CHemische Industrie (BBCH) development scale [33], the initial phase of ripening (BBCH 80-89) occurs in the apparent absence of ethylene production (Phase 1). This is similar to the progression of ripening observed in ‘non-climacteric’ fruit such as grape. However, although no ethylene production is detected during Phase 1 ripening, the ethylene inhibitor 1-Methylcyclopropene (1-MCP) can delay the rapid softening effect, suggesting a response to basal ethylene levels in the fruit [39]. Once the fruit are soft (<10 N), there is a second ripening phase (Phase 2) in which autocatalytic ethylene production is associated with significant increases in volatile ester and terpene synthesis [34, 35] and senescence (BBCH 90-92) [33]. Hence, even though kiwifruit are largely referred to as an autocatalytic ethylene responsive fruit (climacteric) [40, 41], physiological evidence shows that kiwifruit behave differently from a typical climacteric fruit, such as avocado, banana or tomato. Instead, it should be considered towards one end of a non-climacteric – climacteric continuum, where ethylene production occurs at the end of the ripening process.

Genomic technologies such as Expressed Sequence Tag (EST) sequencing [42], molecular maps [43] and transformation protocols [44] have greatly assisted in understanding of *Actinidia* species at the molecular level. A number of studies have identified individual members of the ripening-associated ethylene biosynthetic genes *ACO, ACS* and *SAM* synthase [8, 45, 46], and ethylene signalling components, including *CONSTITUTIVE TRIPLE RESPONSE* (*CTR*), *ETHYLENE RESPONSE SENSOR* (*ERS*) and *ETHYLENE RESPONSE 1* (*ETR1*)-like genes [47]. In this study, we utilised genomic information from *A.chinensis* ‘Hongyang’ [48] to identify ethylene-related genes and known controllers of fruit ripening, and investigated their expression at key time points over fruit maturation and ripening, to better understand the unusual ripening process observed in kiwifruit.

## Results

### Characterisation of maturation and ethylene responses in *A. chinensis*

**Fig. 1:**
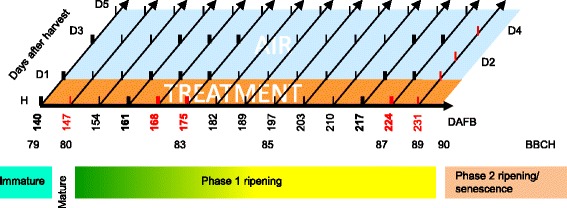
Two dimensional developmental stage X response time schema. Developmental stage/Dimension 1: Fruit were harvested weekly from 140 to 224 days after full bloom (DAFB). Fruit were assessed for a range of physiological parameters immediately at harvest (H). Developmental stage is given using the BBCH stage described in [33]. Immature fruit (BBCH 79) contained 80 % black seeds, and 100 % at BBCH 80 and all following stages. Phase 1 ripening (BBCH 80-89) occurs in the apparent absence of ethylene production. Phase 2 ripening (BBCH 90-92) is associated with autocatalytic ethylene production, volatile ester and terpene synthesis and senescence. Response time/Dimension 2: After harvest, fruit were treated with either propylene, ethylene or stored in air for 24 h. Fruit were then transferred to air and assessed for a range of physiological parameters at the points indicated by vertical bars between 1 and 5 days after harvest (DAH). Tissues selected for a mRNA-seq screen are indicated with a red text/bar and those selected for qPCR with a bold text/bar

**Fig. 2:**
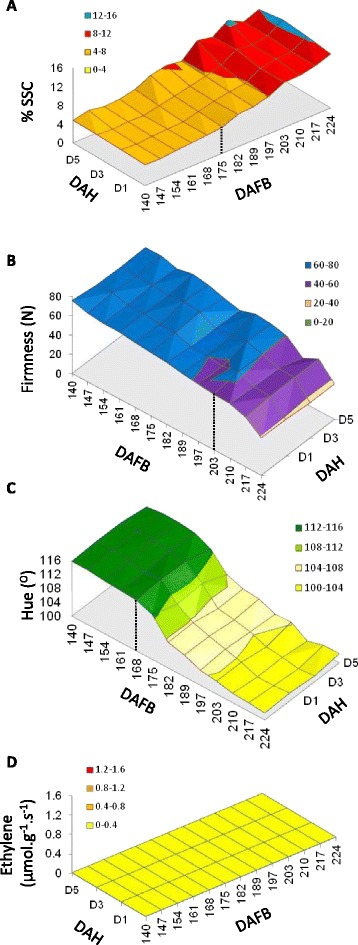
Physiological changes and ethylene production in kiwifruit stored in air during late maturation and Phase 1 ripening. Fruit were harvested from 140-224 days after full bloom (DAFB; BBCH stages 79-87) and stored in air for 1, 3 or 5 days after harvest (DAH). **a** Soluble solids content (SSC), **b**. Fruit firmness, **c**. Flesh colour, **d**. Endogenous ethylene production. Dashed vertical lines show significant changes in the physiology as described in the text

**Fig. 3:**
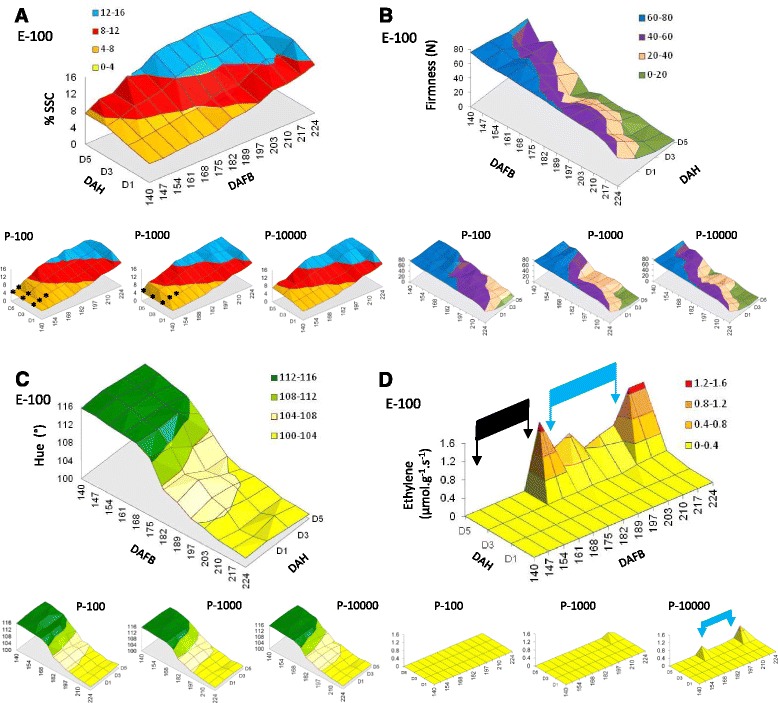
Physiological changes in kiwifruit during late maturation and Phase 1 ripening in response to ethylene and propylene treatments. Fruit were harvested from 140-224 days after full bloom (DAFB; BBCH stages 79-87) and treated for 24 h with ethylene (100 μL.L^-1^; E-100) and different concentrations of propylene (P-100, P-1000 and P-10000 in μL.L^-1^) and assessed 1, 3 and 5 days after harvest (DAH). Black stars represent interpolated data points due to missing data. **a** Soluble solids content (SSC), **b**. Fruit firmnesss, **c**. Flesh colour, **d**. Endogenous ethylene production. The black bar (140-161 DAFB) indicates a period when ethylene production was not detected. The blue bar (182-210 DAFB) indicates a period when ethylene production was repressed

A two dimensional developmental stage X ethylene response time study of *A. chinensis* ‘Hort16A’ fruit was conducted by sequentially harvesting fruit at weekly intervals from 140 days after full bloom (DAFB) to 231 DAFB (Fig. 1). This time frame was chosen to cover kiwifruit maturation and Phase 1 ripening as described previously by Richardson et al. [33] from a mature fruit at stage 79 on the BBCH scale (80 % black seeds) to fruit undergoing on-vine softening at stage 89 (30 N firmness). Each week, a batch of 20 fruit was assessed at harvest (H) for physiological attributes such as soluble sugar content (SSC), outer pericarp colour, firmness, and ethylene emission (Fig. 2). Each week, four batches of 20 fruit were also treated with one of three concentrations of the ethylene analogue propylene (low-100; medium-1000 and high-10,000 μL.L^-1^) and ethylene (100 μL.L^-1^), for 1 day and then transferred into air. As a control, 20 fruit were left untreated. Physiological attributes of treated and untreated fruit were assessed 1, 3 and 5 days after harvest (Figs. 2, 3 and Additional file 1).

Fruit harvested over the 14-week period showed a well documented progression of ripening, with fully black seed observed at 147 DAFB (BBCH 80, as described in [33]). Flesh colour change from a green hue angle (116°) to yellow (100°) started at 168 DAFB, with the majority of colour change occurring by 182 DAFB (Fig. 2c). Soluble sugars started increasing at 175 DAFB (BBCH 83; Fig. 2a), while the start of softening occurred at 203 DAFB, and rapidly increased at 224 DAFB (BBCH 87; Fig. 2b). During the experiment no detectable endogenous ethylene production was measured (Fig. 2d). At each time point, for each of these attributes, fruit kept for 5 days in air (with no ethylene or propylene) at ambient temperatures showed little further ripening progression from fruit measured at harvest (Fig. 2).

When treated with propylene or ethylene, at 140 DAFB, all treatments induced a small increase in SSC 5 days after harvest, from 4.5 % to 8 % (Fig. 3a, Additional file 1). As the fruit matured, SSC increased in response to ethylene and propylene, with a maximum fold-change, between harvest and 5 days after treatment, at 175 DAFB. At the beginning of the time course (140 DAFB), the fruit did not soften when treated with ethylene or propylene. A small drop in firmness, was observed following a one-day treatment of high propylene or ethylene, for treatments at 147 DAFB. The amount of ethylene-induced softening increased as the fruit matured, with a maximum softening response first observed in samples harvested at 175 DAFB. Interestingly, fruit harvested subsequently (between 175 DAFB and 210 DAFB), displayed a reduced softening response to ethylene/propylene. The maximum softening response to ethylene was again observed for fruit harvested after 210 DAFB (Fig. 3b). Softening displayed dose dependent response, with lower concentrations of propylene showing reduced softening. As the fruit progressed through Phase 1 ripening, less propylene was required to achieve a full softening response. Finally, the propylene and ethylene treatments resulted in only a small effect on the rate of colour change within the five-day assessment period (Fig. 3c).

No endogenous ethylene was produced by fruit after propylene or ethylene treatments until 175 DAFB (black bar, Fig. 3d). A significant amount of endogenous ethylene was produced (compared to untreated fruit, *P* < 10^-6^) at this developmental point, five days after an ethylene or high propylene treatment. The amount of ethylene produced (5 days after treatment) between 182 and 210 DAFB was significantly lower (*P* < 0.001 at 182 DAFB, *P* < 0.01 at 189 DAFB, not significant at 197 DAFB, P < 0.001 at 203 DAFB and *P* < 10^-6^ at 210) (blue bar, Fig. 3d). Higher amounts of endogenous ethylene were again produced at 217 and 224 DAFB. The reduced ethylene production observed between 182 and 210 DAFB was consistent with a reduction in softening observed in the same period (Fig. 3b). This result was also seen in batches of fruit treated with high doses of propylene (Fig. 3d). This observation suggests that following maturation there is a developmental progression in Phase 1 ripening through three stages; a start when the fruit are competent to respond to ethylene in a reduced manner but do not produce ethylene (147-175 DAFB), a period where endogenous ethylene production is repressed (to 210 DAFB), and finally a late stage after which endogenous ethylene production is not repressed (217 DAFB onwards).

### Alignment of physiological changes with development associated genes

Physiological changes that were observed during maturation and the three stages of Phase 1 ripening, were aligned with the expression of five previously reported ripening-associated genes [33]. RNA was extracted from fruit at 140 DAFB when no ripening-associated responses were observed (except a small increase in SSC); from fruit at 161, 168, and 175 DAFB corresponding to the development of competency to respond to ethylene, and the breakdown of stored starches and finally from fruit at 217 and 224 DAFB, with the start of on-vine softening. The treatment-time points selected were at harvest, after 24 h high propylene treatment (1 day after harvest), and 2 days later (3 days after harvest) (see schema in Fig. 1).

**Fig. 4:**
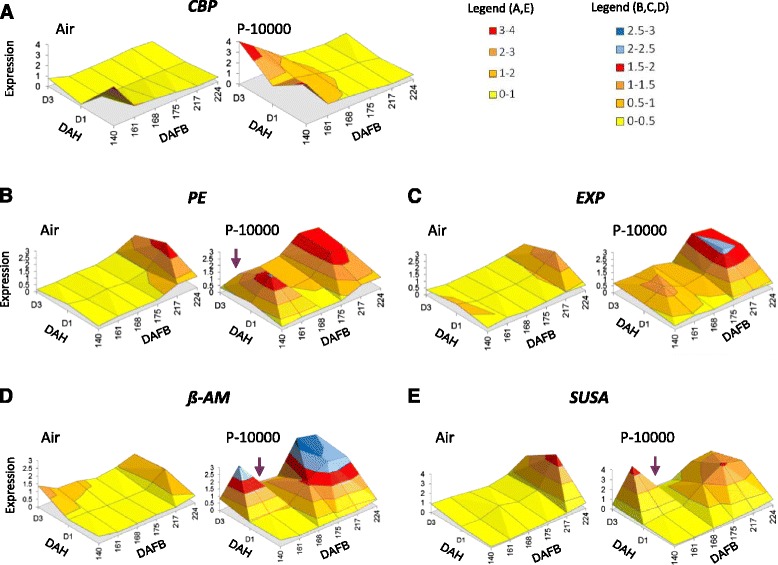
Relative expression changes of five selected kiwifruit genes in response to propylene treatments during late maturation and Phase 1 ripening. Fruit were harvested from 140-224 days after full bloom (DAFB) and left in air or treated with 10,000 μL.L^-1^ propylene (P-10000) for 1 day. Expression was determined at harvest, and 1 day and 3 days after harvest (DAH) using gene-specific primers for **a**. *CBP: CHLOROPHYLL BINDING PROTEIN* (FG05168), associated with colour change; **b**. *PE: PECTIN ESTERASE* (Achn064451) and **c**. *EXP: EXPANSIN* (FG442832), both associated with softening and **d**. *β-AM: β-AMYLASE* (Achn387071) and **e**. *SUSA: SUCROSE SYNTHASE* (Achn024141), both associated with sugar metabolism. Mean expression values are given relative to *AcACTIN*, using two independent biological replicates repeated four times. Arrows show periods where expression is repressed

In the ‘at-harvest’ samples through the maturation period, there were only small transcriptional changes observed in two soluble sugar-related genes (*β-AMYLASE* (*β-AM*) and *SUCROSE SYNTHASE (SUSA)*) and the two softening-related genes (*PECTIN ESTERASE* (*PE*) and *EXPANSIN* (*EXP*)) (Fig. 4, Additional file 2). The colour-related gene *CHLOROPHYLL BINDING PROTEIN* (*CBP)* showed a decrease in expression through Phase 1 ripening. In fruit that were not treated with propylene the effect of harvest over three days was initially minimal, but at 224 DAFB the sugar and cell wall related genes showed an increase in expression 1-3 days post harvest.

Consistent with the physiology, all genes tested except *CBP,* showed no change in expression after propylene treatment at 140 DAFB. From 161 DAFB onwards, a range of expression changes were observed in response to propylene treatment. The soluble sugar-related genes *SUSA* and *β-AM* showed similar expression patterns with up-regulation at 3 days after harvest (DAH) in 161 DAFB fruit, no response at 168 DAFB, transient up-regulation at 1 DAH for fruit at 175 DAFB, and a sustained up-regulation at both 1 DAH and 3 DAH in fruit from 217 DAFB onwards. With the early ripening genes *PE* and *EXP*, there was no response at 140 DAFB, a transient up-regulation at 1 DAH for fruit at 161 DAFB, no response at 168 and 175 DAFB, and a sustained up-regulation at 1 DAH and 3 DAH from 217 DAFB onwards (Fig. 4).

### Identification of genes associated with ethylene biosynthesis and transduction

**Fig. 5:**
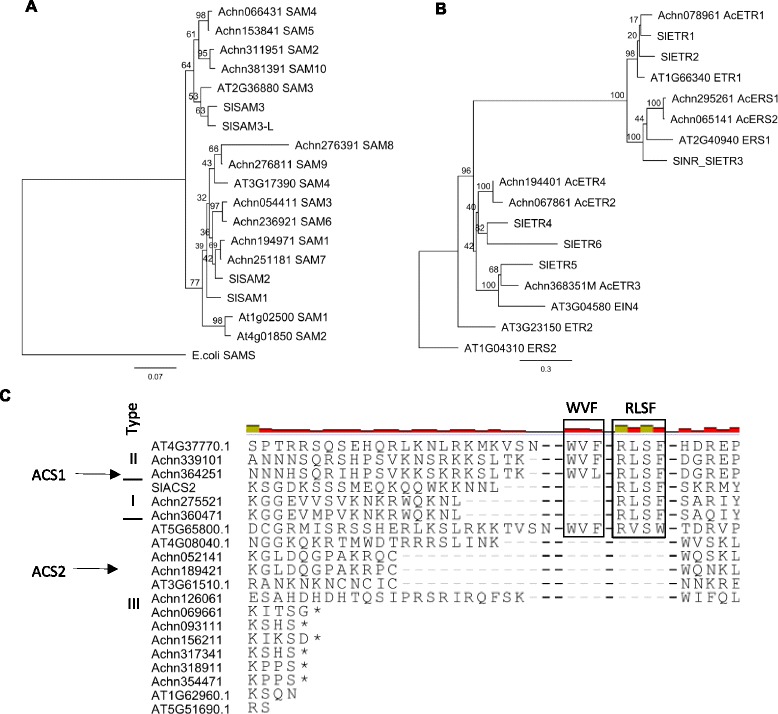
Identification of kiwifruit genes associated with ethylene biosynthesis and transduction. **a** Phylogenetic tree of kiwifuit SAM synthetase showing bootstrap values calculated on 1000 substitutions. SAM synthetase from *Escherichia coli* (CAH56923), *Solanum lycopericum* SlSAM1 (CAA80865), SlSAM2 (CAA80866), SlSAM3 (NP_001234004), SlSAM3-L (XP_010312254). **b** Phylogenetic tree of kiwifruit ethylene receptors showing bootstrap values calculated on 1000 substitutions. Ethylene receptors SlETR1 (U41103), SlETR2 (AF043085), SlNR (U38666), SlETR4 (AF118843), SlETR5 (AF118844), SlETR6 (AY079426). **c** Alignment of the C-terminal region of kiwifruit ACS proteins with Arabidopsis (AT) ACS proteins and the type II autocatalytic ethylene tomato protein SlACS2 (P18485). RLSF and WVF domains are highlighted. Arrows indicate ethylene induced genes

As ethylene production and perception is central to the response-time dimension, we undertook a detailed study to identify all the ethylene biosynthetic and signal transduction genes in the kiwifruit genome [48]. Using lists of the auto-annotated genes we searched for descriptors associated with ethylene biosynthetic and transduction pathways (Table 1). Ten annotated *SAM* synthetase genes were identified, only one of which has been previously identified (Fig. 5a) [46]. Thirteen *ACC SYNTHASE*-like genes were selected, of which only one (*ACS1*) has been published [45, 46]. In other species the ACS proteins have been divided into three classes (I-III), depending on presence of phosphorylation sites (Class I, II) and a WVF and RLSF C-terminal TOE (Target of ETO1) domain (Class II) which confers instability to the proteins. Three ACS proteins contained just the RLSF (Class I), one had both domains (Class II), and nine had neither of these domains (Class III) (Fig. 5c). ACS proteins in Class II are rapidly degraded through the action of an F-Box protein ETHYLENE OVER PRODUCER (ETO); and four *ETO-*like genes were identified in the kiwifruit genome. There were ninety-four *ACC OXIDASE* annotated genes, nine of which have been published [8]. Alignment of the protein sequences of these gene models suggested that 54 had a primary structure similar to *ACO*-like genes.

**Table 1:** Numbers of *A.chinensis* genes associated with ethylene biosynthesis and transduction annotated in the ‘Hongyang’ genome (for gene names see Additional file 4)

Name	Numbers of predicted genes
SAM SYNTHASE	10
ACC SYNTHASE	13
ACC OXIDASE	54
Ethylene receptors	9
EIN3	7
ETO	4
ERF/AP2	209

In the ethylene detection and signal transduction pathway, nine annotated ethylene receptors were identified in the annotated kiwifruit gene models, four of which have been published [45] (Fig. 5b). Further analysis showed that two of these models did not have the all the receptor domains and one was truncated, suggesting these were not functional receptors. Ethylene receptors fall into four main classes of genes, these include the *ETHYLENE RESPONSE1* (*ETR1*), *ETHYLENE RESPONSE SENSOR1*/*NEVER RIPE* (*ERS1*/*NR*), *ETHYLENE INSENSITIVE4* (*EIN4*) and *ETHYLENE RESPONSE2 ETR2-*like genes. Kiwifruit had representative genes in all these gene classes. The transcriptional control of the ethylene signal is through *ETHYLENE INSENSITIVE3 (EIN3*) and *ETHYLENE RESPONSIVE FACTOR/APETALA2* (*ERF*/*AP2*) class of genes. In the annotated kiwifruit genes seven *EIN3*-like genes were observed, and 209 *ERF/AP2*-like genes, of which 182 had a conserved DNA binding domain.

### Identification of ripening associated genes using a mRNA-seq screen and expression analysis of selected genes

Genes involved in ethylene biosynthesis, ethylene response but also transcription factors involved during fruit maturation and ripening belong to large multigene families (Table 1). To select specific ethylene associated genes to analyse further, we used an RNA-seq screen to identify genes within the 2 dimensional space, by selecting time points along the two boundaries. In the ethylene response time dimension, samples were harvested from fruit at 231 DAFB after exogenous treatment with 100 μL.L^-1^ ethylene for 24 h, immediately following the ethylene treatment (1 DAH), 1 day (2 DAH) and 3 days (4 DAH) following treatment. Along the developmental stage dimension we chose harvest samples through Phase 1 ripening (147, 168, 175, 224 and 231 DAFB). (Figure 1, Additional file 3).

The expression of each of the ethylene biosynthetic and signal transduction associated genes identified in the kiwifruit genome was examined in this screen, and genes that appeared to be upregulated in the ethylene response time dimension at 231 DAFB were identified. Of the ten SAM synthetase genes, three had high (Reads Per Kilobase per Million) RPKM values throughout the experiment (*SAM6*, *SAM7* and *SAM10*), and two appeared to be upregulated with ethylene (*SAM1* and *SAM2*) (Additional file 4). Of the thirteen *ACS* genes, two were strongly upregulated with ethylene (*ACS1,*belonging to Class I), and *ACS2* belonging to Class III). Of the 54 *ACO* genes, three were strongly upregulated by ethylene (*ACO1*, *ACO2/3* and *ACO5*). The *ERS1* and *ETR2* classes of ethylene receptors also showed upregulation in expression (Additional file 4).

**Fig. 6:**
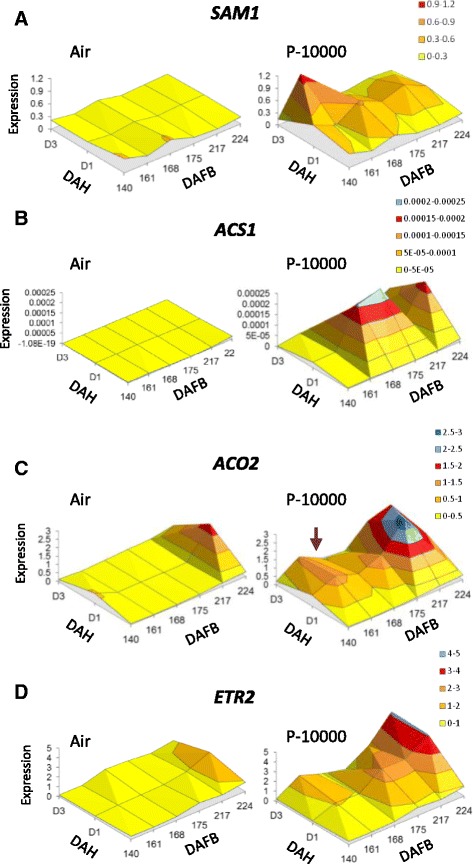
Expression of selected kiwifruit genes associated with ethylene biosynthesis and perception. Fruit were harvested from 140-224 days after full bloom (DAFB; BBCH stages 79-87) and treated in air or with 10,000 μL.L^-1^ propylene (P-1000) for 1 day. Expression was determined at harvest, and 1 day and 3 days after harvest (DAH) using gene-specific primers for: **a**. *AcSAM1: S-ADENOSYL METHIONINE SYNTHETASE1* (Achn194971), **b**. *AcACS1: ACC SYNTHASE1* (Achn364251), **c**, AcACO2: *ACC OXIDASE2* (Achn326461), and **d**, *AcETR2: ETHYLENE RECEPTOR2* (Achn067861). Mean expression values are given relative to *ACTIN*, using two independent biological replicates repeated four times

The expression patterns of four ethylene-related genes with the biggest changes in RPKM values in the response-time dimension in each gene family (*SAM1*, *ACS1*, *ACO2/3* and *ETR2*) were assessed by qPCR at harvest and at time points previously described (Fig. 6, Additional file 5). In fruit at harvest during Phase 1 ripening, *SAM1*, *ACS1* and *ACO2/3* did not increase in expression, while *ETR2* showed an increase during Phase 1 ripening. There was no increase in *ACS1* expression in air treated fruit over the 3 day period, but *ACO2/3* and *ETR2* both showed an increase as the fruit went into the late Phase 1 ripening stage (224 DAFB).

In propylene treated fruit, *SAM1* showed a sustained increase in expression at 140 DAFB, and as the fruit underwent Phase1 ripening (168 – 224 DAFB) a transient increase in expression was observed. *ACS1* showed no response at 140 DAFB and 161 DAFB and a transient increase in expression at 168 and 175 DAFB. There was a minor response at 217 DAFB and then a partial sustained response at 224 DAFB. The *ACO2/3* and *ETR2* genes had more complex responses, with *ACO2/3* showing low transient response at 140 DAFB, a low sustained response at 161 DAFB and 168 DAFB, a transient response at 175 DAFB and a high sustained response at 217 DAFB onwards. *ETR2* had no response at 140 DAFB to ethylene and then low sustained response at 161 DAFB, no response at 168 DAFB, a transient response at 175 DAFB, and an increasing sustained response at 217 and 224 DAFB.

### Regulators associated with competence to ripen

The MADS-box class of genes have been shown to be key regulators of ripening in other fruit species. These include the tomato genes *SlRIN* (*SEPALLATA-*like), *SlTDR4* (*FUL*-like) and *SlTAGL1* (*AGAMOUS*-like). The complex intron/exon structure and alternative splicing of the MADS-box genes makes the automated annotation of this class of genes difficult. The automated prediction of MADS-box genes in the kiwifruit genome is therefore not accurate. Indeed, of the three kiwifruit *SEPALLATA* genes published [33, 49], only the *SEP1* and *SEP2* are annotated in the ‘Hongyang’ genome sequence. Notably, *SEP4* (GB - HQ113364) was absent from the gene models, as was *FUL1* (GB - HQ113357). There were, a further two gene models that showed high similarity to the *SlRIN* DNA binding domain. When examined in more detail, these Ac*SEP*-like genes showed lower homology to SlRIN across the whole protein sequence, with the *SEP4* gene displaying the highest homology (64 % identity) throughout the entire gene (Additional file 6). A scan of the mRNA-seq expression data for all genes with a computer assigned MADS-box function, identified a fourth MADS-box gene (*Achn135681*) with a large change in expression during maturation. This gene was closest to the *APETALA3* (*AP3*) class of MADS-box genes.

**Fig. 7:**
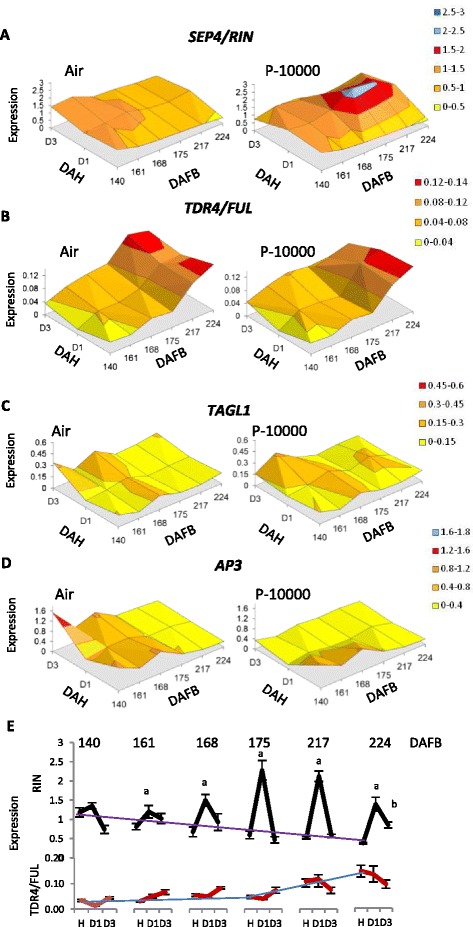
Relative expression changes of MADS box genes in response to propylene treatments during late maturation and Phase 1 ripening. Fruit were harvested from 140-224 days after full bloom (DAFB; BBCH stages 79-87) and treated in air or with 10,000 μL.L^-1^ propylene (P-1000) for 1 day. Expression was determined at harvest, and 1 day and 3 days after harvest (DAH) using gene-specific primers for: **a**. *SEP4/RIN* (HQ113364), **b**. *TDR4/FUL* (HQ113357), **c**. *TAGL1* (Achn121201), **d**. *AP3* (Achn135681). Mean expression values are given relative to *ACTIN*, using two independent biological replicates repeated four times. **e** A comparison of *SEP4/RIN* response to P-10000 treatment and *TDR4/FUL. SEP4/RIN* showed a decrease in expression through maturity (purple line) and a transient upregulation with propylene. TDR4/FUL has little increase in expression until 175 DAFB, then increases (blue line). It has no significant response to propylene. Significance response to propylene compared to harvest time point (a, b = *p* < 0.01)

The expression of the closest kiwifruit *RIN/FUL/TAGL1-* and *AP3-* like MADS-box genes over Phase1 ripening, with and without propylene showed that the MADS-box gene *SEP4/RIN* showed a decrease in expression as the fruit progressed through Phase 1 ripening, and early in Phase 1 ripening (147-168 DAFB) showed little response to the propylene treatment. However after 175 DAFB there was a transient 4-fold up-regulation in expression with the propylene treatment. This transient increase continued to 3 DAH as the fruit went into rapid softening (224 DAFB) (Fig. 7a, e). *FUL*-like increased four-fold as the fruit matured (Fig. 7b/e). When the fruit were treated with propylene there was no difference in this induction, showing this gene acts independently of ethylene. At early Phase 1 ripening (161-168 DAFB) the *TAGL1* gene showed a two-fold increase in expression with an propylene treatment and following harvest. However this increase was not observed later in Phase 1 ripening (Fig. c, Additional file 7). The *AP3*-like gene was highly expressed at 140 DAFB and had a 4-fold decrease in expression as the fruit entered Phase 1 ripening. When treated with propylene this gene was rapidly downregulated (Fig. 7d, Additional file 7).

### Transcriptional control of the SEP4/RIN like gene

**Fig. 8:**
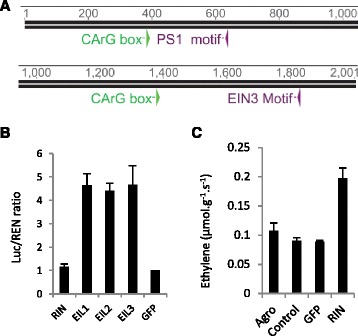
**a** The 2 kb region upstream of the transcriptional start of the *SEP4/RIN* gene showing potential EIN3 binding sites (including a putative PS1 binding site) and MADS box binding CaRG box consensus sequences. **b** Transient transactivation of a 3.2 kb RIN promoter fragment by RIN and EIN3-like transcription factors in tobacco (*N. benthamiana*) leaves with *SEP4/RIN*gene (*RIN*) and three *EIN3* like genes (*EIL*) compared to a *35S:GFP* control (*GFP*). **c** Averages of Ethylene emission from three independent experiments of *A. eriantha* fruit transiently expressing a *35S:SEP4* (*RIN*) gene compared to untransformed *Agrobacterium tumefaciens* (Agro), water (control) and *Agrobacterium* containing a *35S:GFP* gene (GFP)

A 3.2 kb region upstream of the transcriptional start, was firstly scanned for potential RIN-like CaRG sequences. In tomato the preference RIN binding site is CCA(A/T)(A/t)(A/T)ATAG, but RIN can also bind to a more general C(C/T)(A/T)6(A/G)G CaRG box [50]. In the kiwifruit genome sequence there were two CaRG sequences within the first 2 kb of the SEP4/RIN promoter. Also within this promoter is a potential EIN3 binding site (A(T/C)G(A/T)A(C/T)CT), as well as a PS1 sequence that EIN3 has been shown to bind to [51] (Fig. 8a). To test whether these sites are functional, the 3.2 kb fragment was inserted into a Luciferase reporter construct [52] and transiently co-injected into *Nicotiana benthamiana* leaves with the kiwifruit *SEP4/RIN* gene driven by a 35S promoter. It was found that SEP4/RIN was unable to transactivate the RIN promoter. When the 3.2 kb promoter fragment was co-infiltrated with three kiwifruit EIN3 transcription factors driven by a 35S promoter, it was shown that the ethylene associated EIN3-like genes could transactivate the promoter (Fig. 8b).

### The SEP4 RIN-like gene induces ethylene production in fruit

In other species, there has been a strong association between the MADS-box gene *RIN* and ethylene biosynthesis. For example, the *SlRIN* gene binds and activates itself and *SlACS2* [50]. Because of the close similarity with *SlRIN*, and expression pattern, the construct containing the *SEP4/RIN* gene driven by a 35S promoter was tested transiently in mature *A. eriantha* fruit [53]. *A.erinantha* fruit were chosen as they are amenable to transient expression, and produce very low amounts of ethylene at ripening [54]. Transiently injecting *Agrobacterium* containing the 35S:*AcSEP4/RIN* construct into mature fruit produced a consistent increase in endogenous ethylene compared with three controls, water injection, *Agrobacterium* alone and *Agrobacterium* containing a 35S:*GFP* (Green Fluorescent Protein) gene construct (Fig. 8c).

## Discussion

**Fig. 9:**
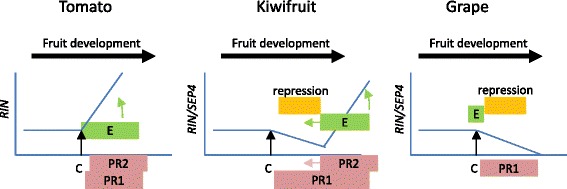
A simplified model of the role of RIN-like genes during fruit maturation and ripening in different scpecies. RIN expression (blue line) correlates with ethylene production (E). Once the fruit is competent to ripen (C) RIN can induce ethylene dependent ripening (Phase 2 ripening PR2), but in the absence of ethylene (and RIN), ethylene independent ripening progresses (Phase 1 ripening PR1). In tomato RIN activates both ethylene and itself (Alba et al. 2005, Fujisawa et al. 2013) [29, 56] progressing PR1 and PR2 ripening simultaneously, while in kiwifruit and grape there is a down regulation of RIN expression following competence to ripen (and in grape veraison) (Pilati et al. 2007) [57] allowing PR1 ripening to occur independently. In kiwifruit this repression can be reversed later in maturity, or with the application of ethylene. Kiwifruit thus shows a hybrid ethylene independent-dependent mechanism with phase 2 ripening being likely to be controlled by SEP4/RIN

During development in all fruits, there is a period when the fruit becomes competent to ripen. This is a major change in the fruit’s properties, as it alters the fruit to be more attractive to seed-dispersing organisms, and usually coincides with seed maturation. The switch is often facilitated by large changes in phytohormone production, especially ethylene. In model fruit species such as tomato and grape there is growing molecular understanding of how some of the key switches regulate this transition. In tomato, ripening is predominantly regulated by ethylene, whilst in grape ripening ethylene has a relatively minor role. At the molecular level the best studied regulator is the *RIN* MADS-box gene, which acts in a heterotetrameric complex with *FUL*/*TDR4 FUL2* and/or *TAGL1* to bind and activate many genes associated with fruit ripening [21, 26, 28, 29, 50]. *RIN* is highly and constantly up-regulated during fruit ripening in tomato [17], banana [55], strawberry [18], and apple [19], and in tomato the FUL/TDR4 RIN complex has been shown to bind and activate the promoter of the ethylene biosynthesis genes *ACS2*,*4* and *RIN* itself [26, 28, 29]. In this study we have shown that a *RIN*-like gene (previously published as *AcSEP4* [33, 49] is associated with autocatalytic ethylene-associated ripening. Unlike tomato *RIN* [56], but consistent with grape *VviSEP4* (GSVIVG0101051001) [57], following maturation (or veraison) the expression of this kiwifruit *RIN*-like gene decreases (Fig. 7b). This suggests that in grape and kiwifruit RIN cannot auto-activate itself, and even suggests the presence of an additional repression during this time. Ethylene (or propylene) is able to induce *RIN* expression in tomato and kiwifruit, however in kiwifruit, subsequent removal of ethylene causes this gene to be downregulated again (Fig. 7b), further supporting a repression mechanism. Transient experiments performed in tobacco indicate that the kiwifruit RIN does not activate *RIN* and there is no increase in *RIN* expression when the *AcFUL* is developmentally turned on, or when *RIN* expression is induced with ethylene, suggesting a mechanism by which the hybrid ripening is controlled (Fig. 9).

While the controllers of kiwifruit Phase-1 ripening (non-autocatalytic ethylene associated) are yet to be fully identified, a key candidate is the *FUL/TDR4*-like gene (*Achn247791*), which in tomato has been shown to be a major controller of many ripening factors [21, 28]. In kiwifruit the increase in *FUL* coincides with the start of rapid softening (Figs. 2 and 7) suggesting a mechanism by which cell wall related genes such as *PE* and *EXP* (Fig. 4) are upregulated. This would be consistent with tomato, where authors have postulated that SlFUL/TDR4 controls an ethylene independent ripening process [21], and our results in kiwifruit would support the suggestion that this occurs independently of *RIN*. In the presence of ethylene (or propylene), these cell wall genes are upregulated, which could be due to the presence of *RIN* or other ethylene-related cis-elements in their promoters. The switch between a transient activation of these genes and sustained activation late in maturity may be explained by the developmentally controlled upregulation of *FUL*. The third kiwifruit gene of the MADS-box complex, *TAGL1* does not appear to change during development during Phase-1 ripening. *TAGL1* is partially upregulated with ethylene similar to tomato [22], but not in a sustained manner (Fig. 7).

In this study a third floral identity-like gene (*AP3*), showed a large decrease in expression as the fruit transitioned into ripening competence. An *AP3* tomato homologue *TDR6* is also expressed early in fruit maturation, is down-regulated at ripening, and interestingly showed a possible interaction with TDR4 (FUL) in a yeast 2-hybrid experiment [58]. Consistent with that of *Arabidopsis AP3*, the expression appears to be auto-inhibitory [23], with a decrease in expression in ripening fruit, and it is inhibited by ethylene. Whether this gene plays a role in the regulation of ripening is yet to be seen.

As the fruit mature there is an increase in sensitivity to ethylene, with the lower concentrations of propylene inducing a more significant response over time (Fig. 1). Each of the ripening responses shows different sensitivities to ethylene and propylene, with sugars showing an early high sensitivity and fruit firmness showing a lower sensitivity. This implies there is a similar sensitivity dependency mechanism occurring in kiwifruit as proposed in apple [59, 60]. Interestingly, kiwifruit colour change shows little effect from the treatment with ethylene, suggesting that de-greening is probably associated with developmental control rather than ethylene. At the transcriptional level, ethylene (propylene) causes a reduction in *CBP* transcription, showing that some colour-related mechanisms are transcriptionally modulated by ethylene. The lack of phenotypic changes may be however partially due to the short 5-day assessment period used in this study.

## Conclusions

With the development of more sensitive detectors, and the use of molecular tools outside the classic model organisms, the distinctions between autocatalytic ethylene associated (climacteric) and non ethylene associated (non climacteric) fruit ripening has eroded [61]. Here we propose a model (Fig. 9) in which, through changes in the regulation of ethylene regulating *RIN*-like genes and ethylene independent *FUL/TDR4* like genes, the differences between fruit ripening between these two classes are not so dissimilar.

## Methods

### Kiwifruit sampling

Yellow-fleshed kiwifruit (*Actinidia chinensis* (Planch.) var. *chinensis* ‘Hort16A’) fruit were sampled from The New Zealand Institute for Plant & Food Research Limited research orchard at Te Puke, Bay of Plenty, New Zealand, during the 2011 harvest season. Sampled fruit were, unless otherwise specified, held in ethylene-free rooms under controlled conditions at temperatures between 18 °C and 20 °C.

### Fruit harvest and assessment

Two batches of 60 fruit each from *A. chinensis* ‘Hort16A’ were harvested at weekly intervals over a 14-week period covering maturation and ripening. Each batch of fruit was independently processed for biological replication and separated into at-harvest samples and five treatments: air, 100, 1000, 10,000 μL.L^-1^ propylene, or 100 μL.L^-1^ ethylene, for 24 h in 20 L sealed containers. Propylene was selected as a viable alternative to ethylene as a treatment for the activation of the ethylene response for two reasons, 1) in order to validate that ethylene detected after treatment was a result of endogenous ethylene production and not confounding from residual treatment gas and, 2) to increase the treatment volume to a amount that could be accurately measured and handled. Air was circulated continuously and lime was used to absorb CO_2_. The fruit were removed and immediately assessed (Day 1 (Day after Harvest - DAH), or were stored for 2 (Day 3) or 4 days (Day 5) in an ethylene-free room at 20 °C and then assessed. Physiological assessments of ten randomly selected fruit from each biological replicate at each time of harvest were assessed. Each fruit was weighed and placed in a 500-mL airtight pot for 45 min, from which ethylene concentrations of 1 mL air headspace were assessed using a Philips PU4500® Gas Chromatograph machine. Destructive fruit assessment was conducted rapidly to reduce wound-associated transcriptional changes. Fruit firmness was determined using a Fruit Texture Analyser (GUSS™ Fruit texture analyser GS-14®, South Africa) with a 7.9-mm probe at 20 mm.s^-1^ following removal of a 1 mm thick slice of skin and outer pericarp at two locations perpendicular to each other at the fruit equator. Flesh colour was determined in an LCH colour space using a CR 300 ChromaMeter (Konica Minolta™ CR-300® Minolta, Japan) with a C65 light source at two locations perpendicular to each other at the fruit equator at a depth of about 2 mm. Fruit were cut in half and the percentage of fully coloured black seeds was recorded. Soluble solids contents were determined from juice taken from both ends of the fruit using a refractometer (ATAGO® ATC-20E, Japan).

### Extraction of RNA for gene expression analysis

Four fruit were selected that displayed the closest firmness to the mean for tissue sampling for expression analysis. Sampled fruit tissue was cut into small pieces, rapidly frozen in liquid nitrogen, and stored at -80 °C prior to further molecular analysis. RNA-seq analysis was performed on selected samples at maturity and also on an additional set of fruit harvested 231 DAFB (softened on vine). This second set of fruit were chosen as they would be fully responsive to ethylene. Fruit were harvested, treated with 100 uL.L^-1^ ethylene, and assessed as above 1 day, 2 days and 4 days following treatment (Additional file 4).

Total RNA was extracted from ~2 g of *A. chinensis* ‘Hort16A’ fruit material as described in [62]. Contaminating genomic DNA was removed from 10 μg of each total RNA sample using an Ambion Turbo DNAse kit according to the manufacturer’s specifications (http://www.lifetechnologies.com). RNA was quantified and quality-assessed using an Agilent 2100 bioanalyzer with a RNA 6000 nanochip. RNA samples with a RNA integrity (Rin) score of >8.0 were used for sequencing. Messenger RNA (mRNA) sequence data were obtained using either NZGL (www.nzgenomics.co.nz) or Macrogen sequencing facility (www.macrogen.com) on an Illumina HiSeq2000 next generation sequencer, with samples bar-coded 12 to a lane, with a sequencing depth of 17-27 million reads (Additional file 4).

For quantitative PCR (qPCR), RNA used for mRNA-seq and a second independent biological replicate were assessed. cDNA was synthesised using an Invitrogen™ VILO kit according to the manufacturer’s instructions (http://www.lifetechnologies.com). Oligonucleotide primers specific to each gene were designed using Vector NTI™ version 11.0 (Invitrogen™) using EST sequences [42]. Primer sequences are found in Additional file 8. For qPCR, four technical repeats of each sample were measure using a 384-well plate LightCycler480™ (Roche™) and a SYBRgreen 480 LightCycler® kit (Roche™). The identity of each amplified fragment was confirmed by sequencing. Differences in gene expression were calculated using two independent reference genes, *ACTIN* and *EF1α* [63], both of which gave similar results, therefore the results presented here are relative to *ACTIN*. Selecting fruit close to the median firmness gave consistent results except for a single time point (168 DAFB fruit treated with 10,000 propylene, assessed after 1 day). Results from this time point were strikingly different and subsequent reassessment suggested one fruit in this batch of four was physiologically an outlier (more advanced for other ripening attributes), so data presented from this time point are from a single biological replicate.

### Bioinformatic processing of mRNA seq data

mRNA-seq data were processed by first removing poor sequence quality from each read using FASTQC version 1.6.0_17. Next, each sequence read was aligned to the *Actinidia* gene models predicted from the kiwifruit genome (http://bioinfo.bti.cornell.edu/kiwi) using Bowtie2 (version 1.0.0) using default settings. Data were transferred to Bioconductor in R and reads were normalised to Reads per Kb per Million (RPKM), using the total number of aligned reads and length of the predicted genes.

### Phylogenetic analysis

Alignments of conserved domains within the proteins were generated using the Geneious Pro™ 7 (www.geneious.com). Multiple alignments were performed using Genious global alignment with free end gaps method. The cost matrix was Blowsum62 and opening penalty of 30 and an extension penalty of 0. The phylogenetic trees were built Geneious Pro™ 7 using PHYML neighbour joining with 1000 bootstraps.

### Promoter analysis

Using genomic sequence from the predicted *A.chinensis* genome, the promoter of the *SEP4/RIN* gene was amplified using two sets of primers. This approach was taken to create a ATG fusion with the *Nco*1 site at the start of the luciferase gene in the pGREEN vector. Primer set 1 (F) 5’Phos GATCCTAAGGTACGCACGATTGAAC and (R) 5’GCTCTCTCTCTCTCTCTCTCTACTGGTA, and primer set 2 (F) 5’CTAAGGTACGCACGATTGAACGTG and (R) 5’Phos CATGGCTCTCTCTCTCTCTCTCTCTACTG. The two 3.2 kb PCR products were melted and reannealed to create *Bam*H1 and *Nco*1 compatible overhangs. This was annealed into the pGreenII 0800-5 LUC vector as described in [52]. Transient activation of the promoter was achieved using a 35S:*SEP4/RIN* construct [49], made by placing the *AcSEP4* gene in front of the 35S promoter using Gateway cloning (Life Technologies Ltd) and 35S:*EIN3* trancription factors described by [47], and tested as described in [52].

### Transient assays

Liquid *Agrobacterium* (strain GV101) cultures containing the 35S:*SEP4/RIN* construct were grown to an OD between 0.75 and 0.85 and activated in Mg_2_SO_4_ (10 mM) with acetosyringone (1 μM) for 30 min. This solution was injected into *A. eriantha* fruit [53] and left for 3 days before assessing for ethylene using the headspace analysis method as described above.

### Statistical analysis

Analysis of physiological data presented in Additional file 1 was calculated by Post-hoc analysis of ANOVA using Tukey’s honest significance test at comparable time points. Statistical calculations for significance of qPCR presented in Additional files 2 and 5 was calculated in a pairwise manner using two tailed Welch’s t-tests. All other statistical calculations were calculated in a pairwise manner using two tailed Student’s t-tests.

## Additional files

Additional file 1:
**Details of physiological changes of sugar accumulation, firmness loss and ethylene biosynthesis in** ***Actinidia chinensis*** **‘Hort16A’ kiwifruit.** (PPTX 1802 kb) Additional file 2:
**Relative expression changes of ripening associated genes in kiwifruit in response to propylene treatments.** (PPTX 98 kb) Additional file 3:
**List of ethylene-related gene expression values mined from** ***A. chinensis*** **mRNA-Seq libraries.** (PPTX 36 kb) Additional file 4:
**Physiology of selected time points for mRNA-Seq, and depth of libraries.** (XLSX 1091 kb) Additional file 5:
**Expression of selected genes associated with ethylene biosynthesis and perception.** (PPTX 103 kb) Additional file 6:
**Assesment of** ***RIN*** **-like genes in** ***Actinidia chinensis*** **‘Hort16A’ kiwifruit.** (PPTX 288 kb) Additional file 7:
**Expression of selected MADS-box genes (RIN, TDR4, TAGL1 and AP3).** (PPTX 136 kb) Additional file 8:
**Sequences of primers used for qPCR.** (PPTX 30 kb)

## Abbreviations

BBCH: Biologische Bundesanstalt, Bundessortenamt und CHemische Industrie
DAFB: Days after full bloom
DAH: Days after harvest
qPCR: Quantitative polymerase chain reaction
RPKM: Reads per Kilobase per Million
