# Supplementary figures and images for: The hybrid non-ethylene and ethylene ripening response in kiwifruit (Actinidia chinensis) is associated with differential regulation of MADS-box transcription factors
Source: BMC Plant Biol. 2015 Dec 29;15:304. doi: 10.1186/s12870-015-0697-9 (PMC4696264; doi:10.1186/s12870-015-0697-9)

## Slide 1
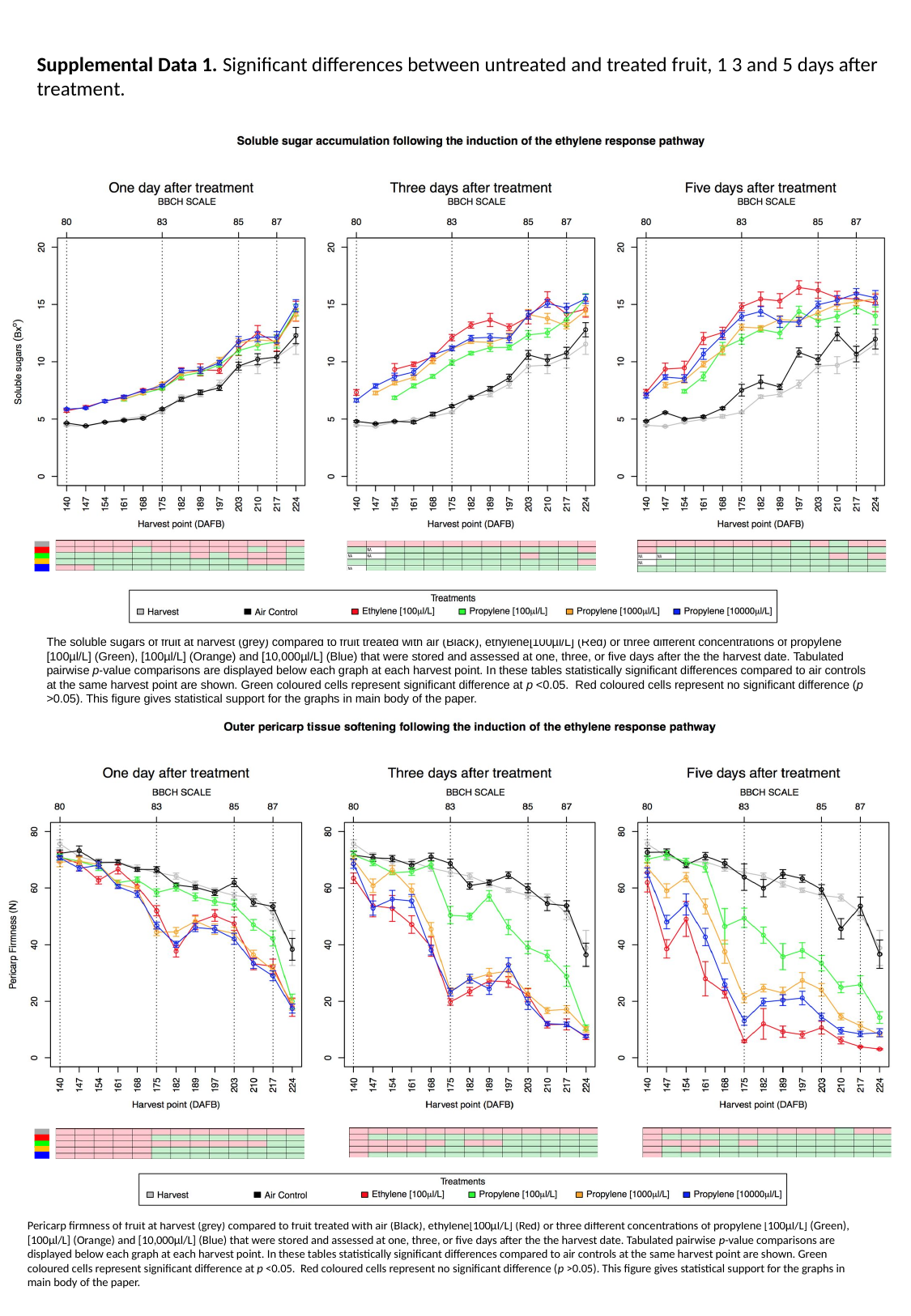

## Slide 2
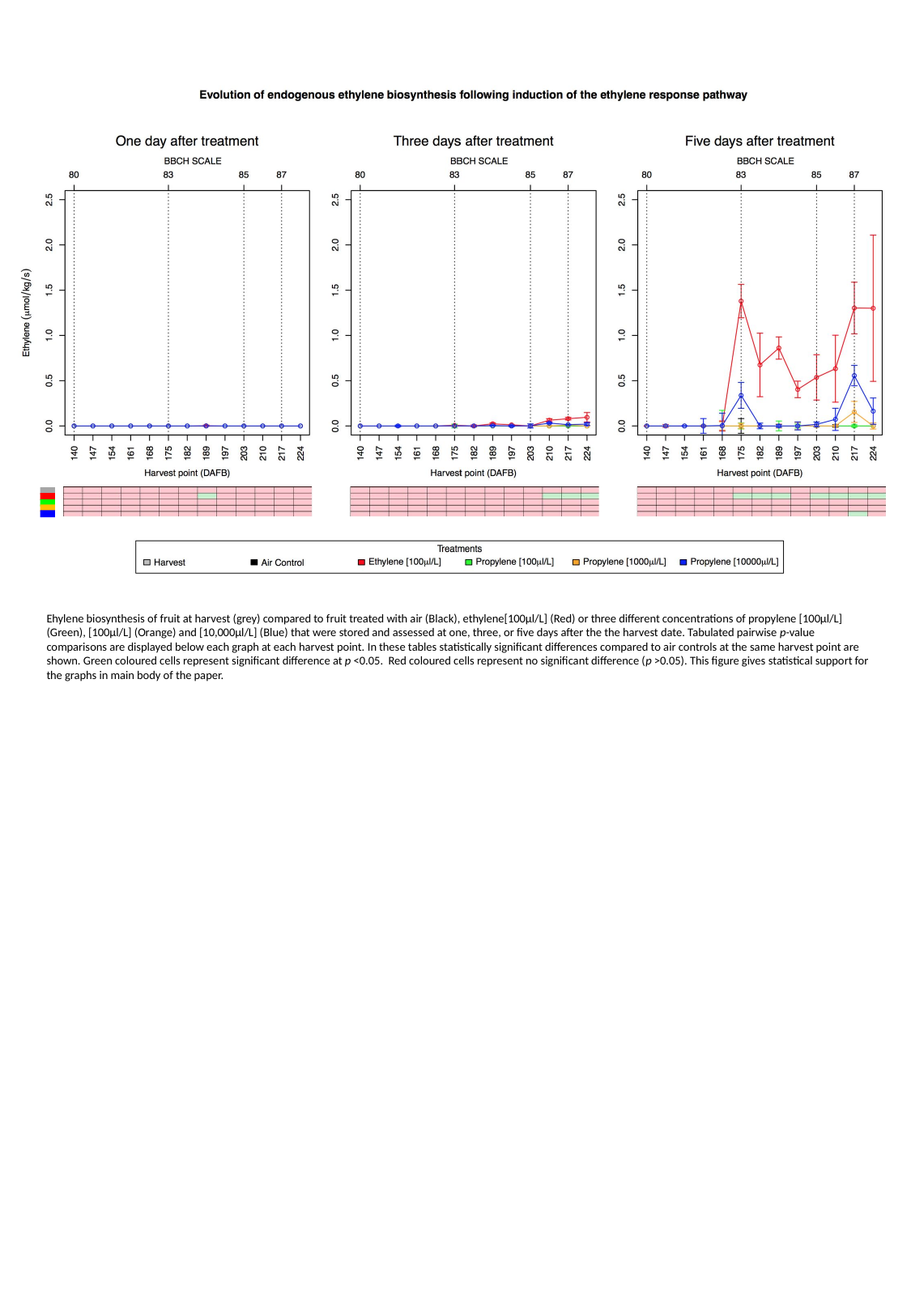

Supplement: Additional file 1: — Details of physiological changes of sugar accumulation, firmness loss and ethylene biosynthesis in Actinidia chinensis ‘Hort16A’ kiwifruit. (PPTX 1802 kb) [file 12870_2015_697_MOESM1_ESM.pptx]

## Slide 1
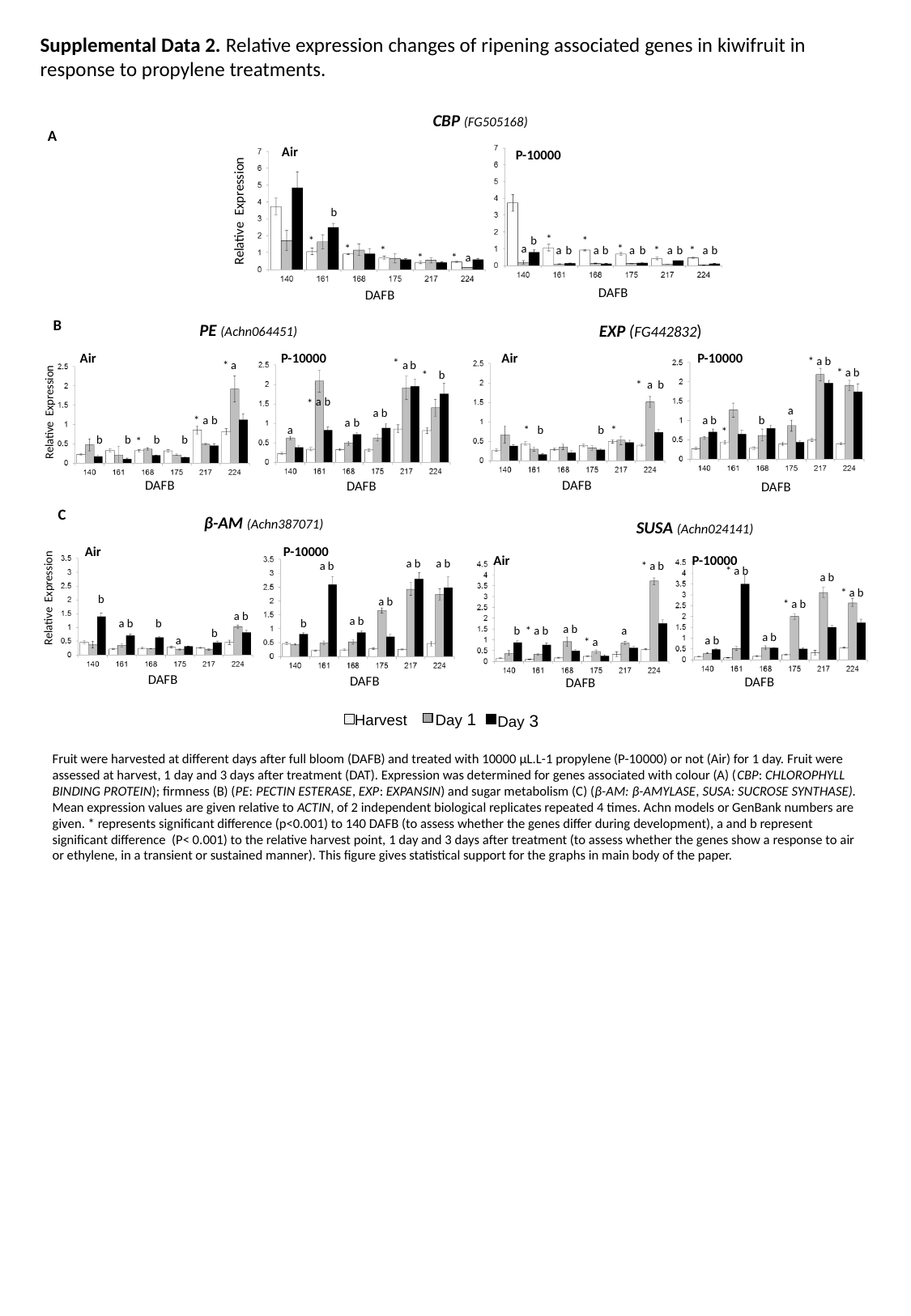

Supplement: Additional file 2: — Relative expression changes of ripening associated genes in kiwifruit in response to propylene treatments. (PPTX 98 kb) [file 12870_2015_697_MOESM2_ESM.pptx]

## Slide 1
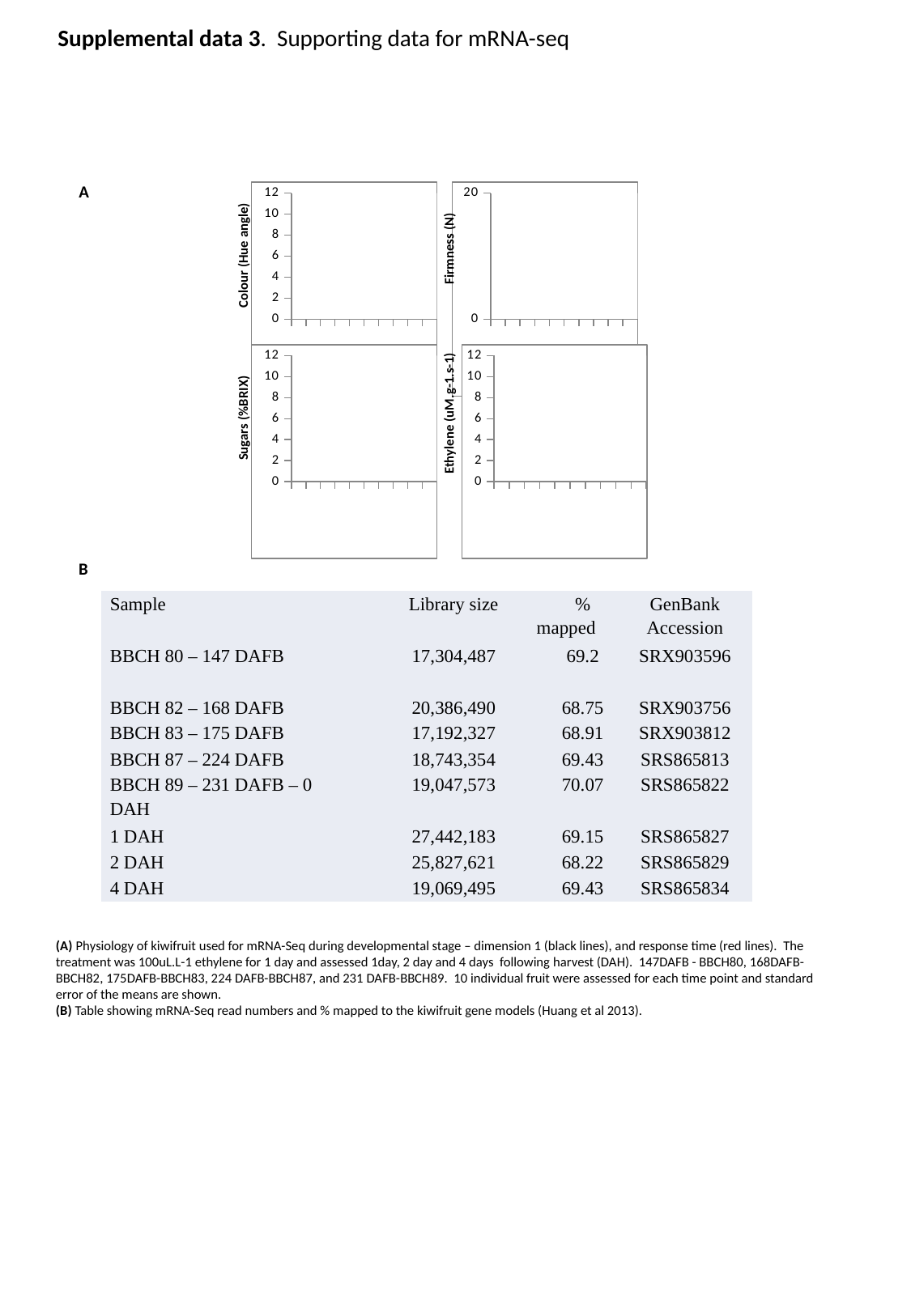

Supplement: Additional file 3: — List of ethylene-related gene expression values mined from A. chinensis mRNA-Seq libraries. (PPTX 36 kb) [file 12870_2015_697_MOESM3_ESM.pptx]

## Slide 1
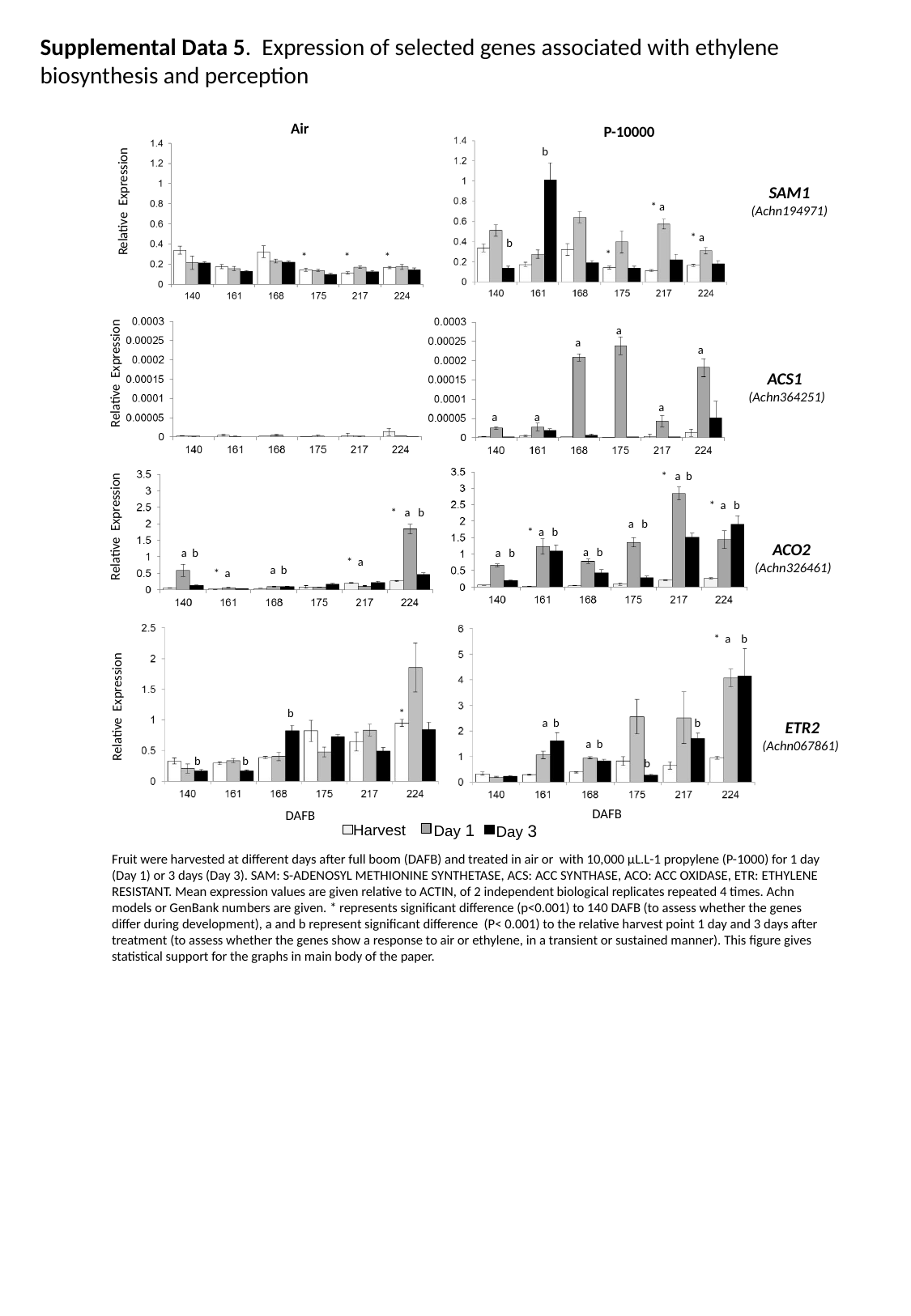

Supplement: Additional file 5: — Expression of selected genes associated with ethylene biosynthesis and perception. (PPTX 103 kb) [file 12870_2015_697_MOESM5_ESM.pptx]

## Slide 1
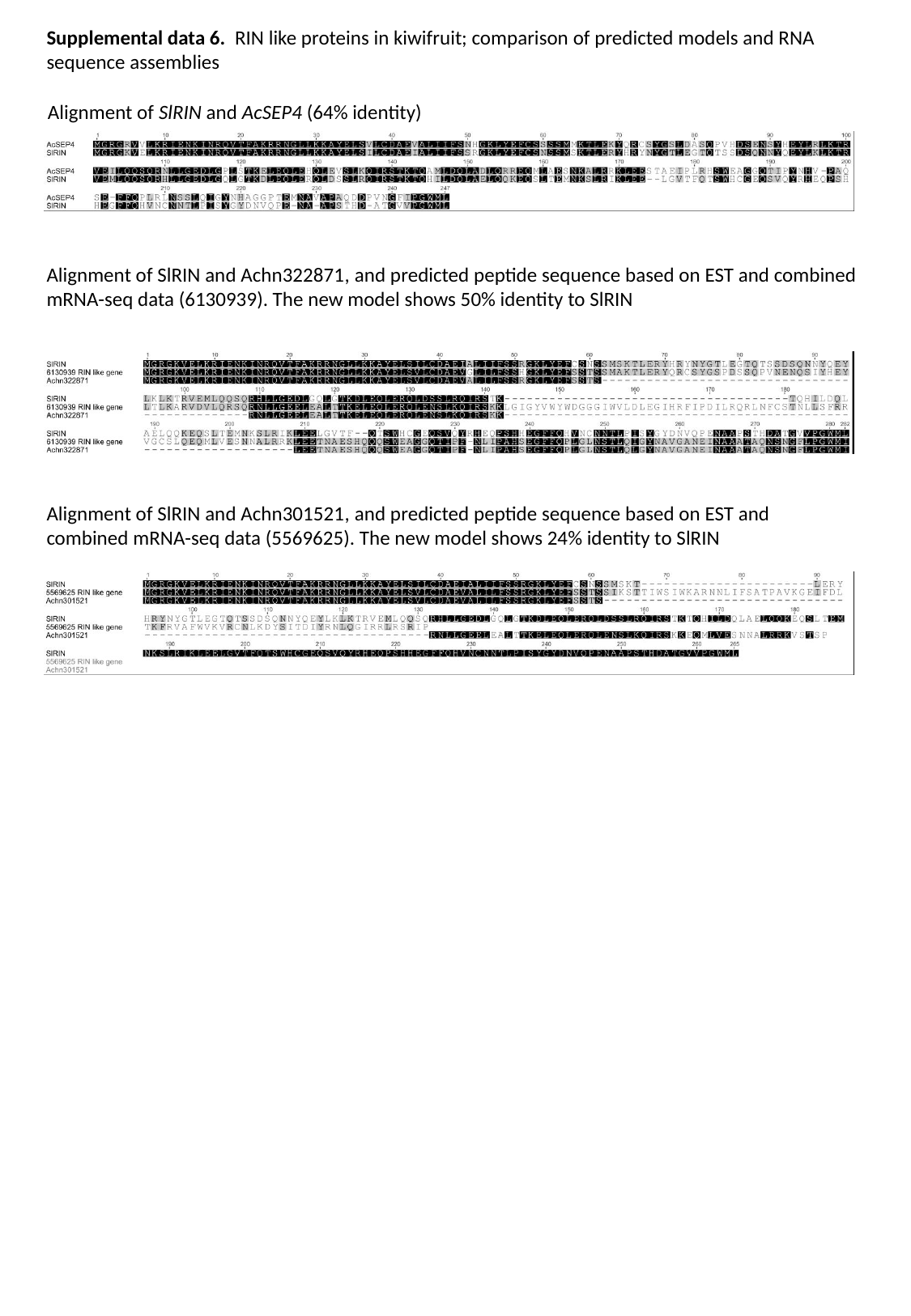

Supplement: Additional file 6: — Assesment of RIN -like genes in Actinidia chinensis ‘Hort16A’ kiwifruit. (PPTX 288 kb) [file 12870_2015_697_MOESM6_ESM.pptx]

## Slide 1
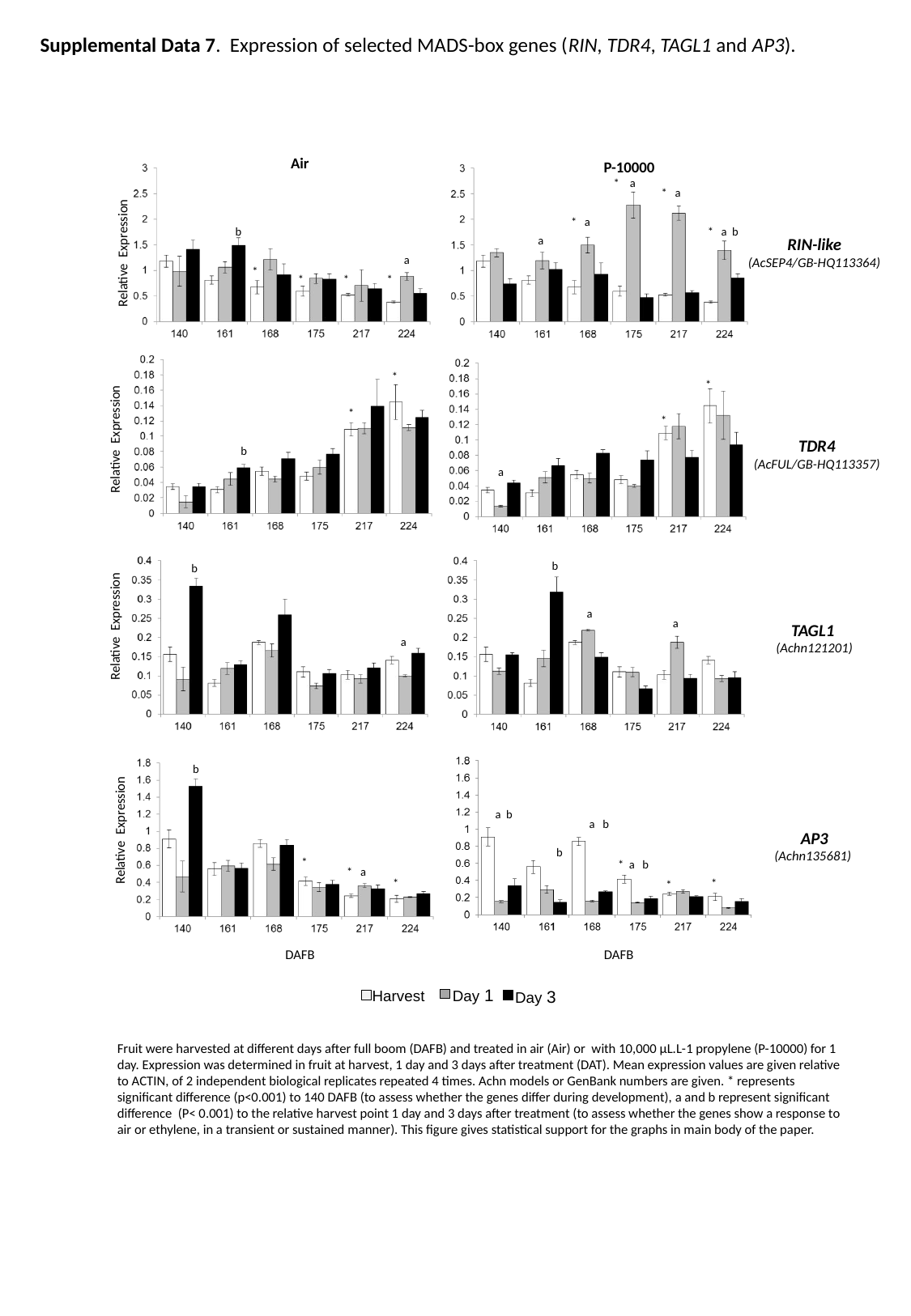

Supplement: Additional file 7: — Expression of selected MADS-box genes (RIN, TDR4, TAGL1 and AP3). (PPTX 136 kb) [file 12870_2015_697_MOESM7_ESM.pptx]

## Slide 1
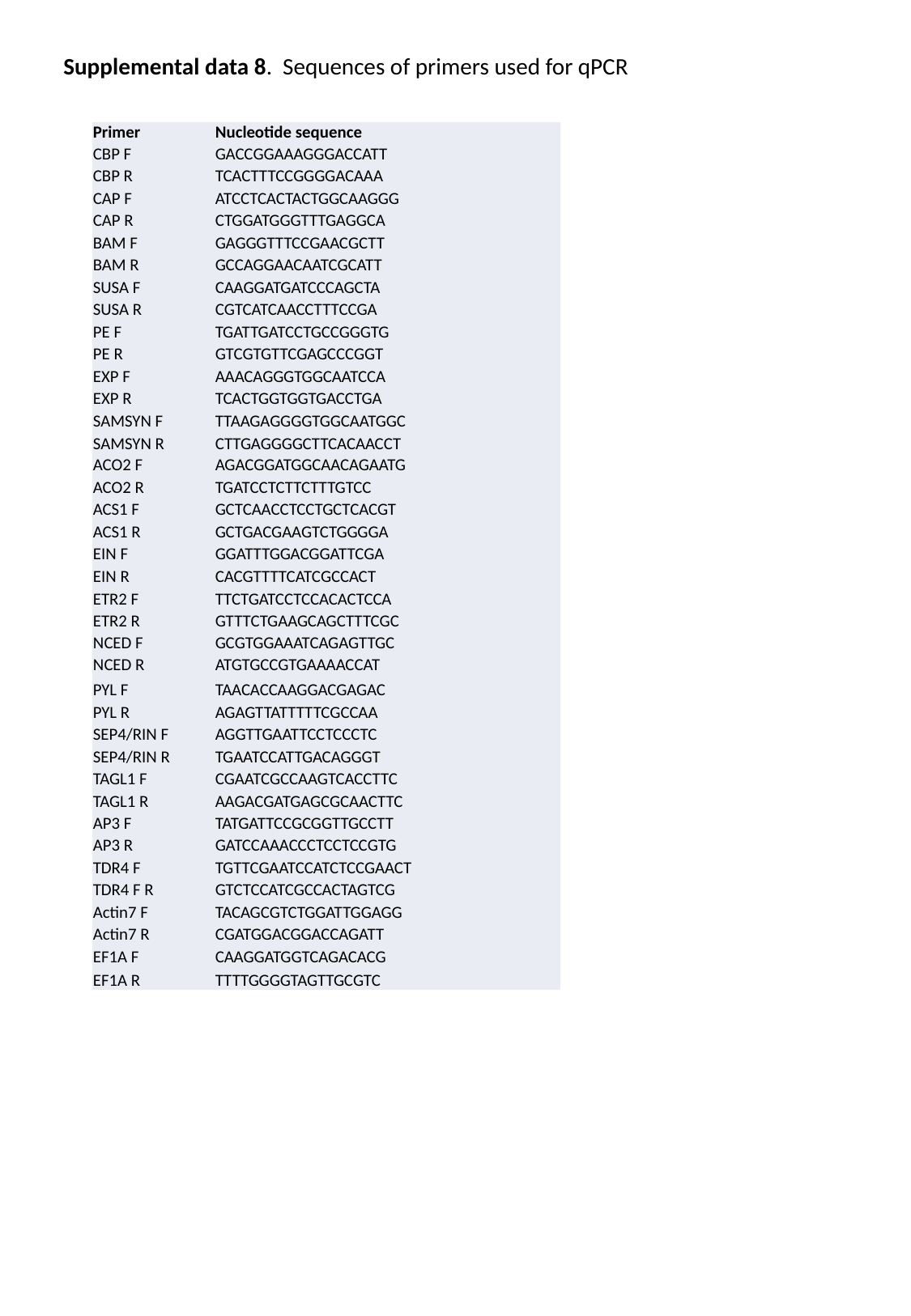

Supplement: Additional file 8: — Sequences of primers used for qPCR. (PPTX 30 kb) [file 12870_2015_697_MOESM8_ESM.pptx]
